# An exploration of reported food intake among inmates who gained body weight during incarceration in Canadian federal penitentiaries

**DOI:** 10.1371/journal.pone.0208768

**Published:** 2018-12-18

**Authors:** Claire Johnson, Jean-Philippe Chaput, France Rioux, Maikol Diasparra, Catherine Richard, Lise Dubois

**Affiliations:** 1 Interdisciplinary School of Health, University of Ottawa, Ottawa, Ontario, Canada; 2 Healthy Active Living and Obesity Research Group, Children’s Hospital of Eastern Ontario Research Institute, Ottawa, Ontario, Canada; 3 School of Nutrition Sciences, Faculty of Health Sciences, University of Ottawa, Ottawa, Ontario, Canada; 4 School of Epidemiology and Public Health, University of Ottawa, Ottawa, Ontario, Canada; McMaster University, CANADA

## Abstract

**Background:**

Canadian penitentiaries have recently been shown to be obesogenic. However, little is known about the eating habits of inmates who gained weight while living in the prison environment.

**Methods:**

This retrospective cohort study examined the reported food intake of inmates during incarceration in federal penitentiaries. During a face to face interview, anthropometric measures (2016–2017) were taken and compared to anthropometric data at the beginning of incarceration (mean follow-up of 5.0 ± 8.3 years). Self-reported data on food intake were collected via a food frequency questionnaire.

**Results:**

Inmates who gained the most weight (15.7 kg) during incarceration reported not eating vegetables. They were followed by inmates who gained 14.3 kg and reported not eating fruit. Other inmates who gained a significant amount of weight reported not eating cereal, dairy or legumes. Moreover, inmates’ weight gain was also assessed by special diets: inmates following a religious diet (4.5 kg) or a diet of conscience (-0.3 kg) gained less weight than inmates not following a diet (5.8 kg). In comparison to other types of diets, inmates on a medical diet gained the most weight (7.5 kg). Furthermore, inmates who gained significant weight (8.0 kg) also reported not purchasing healthy foods from the commissary store (or “canteen”), whereas inmates who gained less weight (4.8 kg) reported purchasing healthy foods from the commissary store (or “canteen”). The observed weight gain was positively associated with food purchased from the commissary store (or “canteen”), but was not associated with the feeding system of the penitentiary (tray, cafeteria or meal plan).

**Discussion:**

Food intake during incarceration is a modifiable risk factor that could be the target of weight management interventions with inmates. Our findings suggest that inmates who gained the most weight also reported having low intake of foods deemed healthy (vegetables, fruit, cereal, dairy and legumes) from food services and from the commissary store (or “canteen”) purchases.

## Introduction

Correctional Service Canada is responsible for feeding all men and women incarcerated in federal penitentiaries in Canada. As such, inmates forfeit much of their control when being fed. This makes food a daily preoccupation for prisoners. In support of that statement, correctional staff, who hear the chatter in prison halls confirm that inmates are often discussing their previous meal or anticipating their next one [1]. As author Rosie Meek wrote, many experts from the correctional service world agree: “There are three things you need to get right in prison: the food, the visits and the gym”[2].

To feed the prison population, Correctional Service Canada has a four week non selective cycle menu that is based on Canada’s Food Guide and provides ~2600 calories per day for male inmates and ~2000 calories per day for female inmates [3]. The budget to purchase food for this standardized menu is ~$5 daily per inmate [3]. In Canada, federal penitentiaries use the menu for two of the three different feeding systems. The menu is used in the central food production system with tray delivery, or with cafeteria delivery. The third system is called small group meal plan program, and inmates on this plan do not follow the standardized menu. In this setting, inmates are responsible for the purchase and preparation of their food. With the latter system, inmates are given a food budget of ~$35 per week and they choose their food items to purchase from the prison “grocery store”. Depending on the food system, inmates may eat alone in their cell (central production and tray delivery), or they may eat with their fellow inmates in a dining hall (central production and cafeteria delivery), or they may prepare and eat their meals in kitchenettes with a few housemates (decentralized production in small group meal plan).

In all penitentiaries, regardless of the feeding system, inmates have access to the commissary store (or “canteen”) where they can purchase foods of their choice with their own funds [4]. The foods available for purchase are chosen by the inmate committee of the penitentiary and approved by management. The list of foods can vary between penitentiaries since it is not standardized by Correctional Service Canada. In addition to the menu, accommodation diets are available to inmates for religious reasons (e.g., kosher diets, halal diets), as part of their medical treatment (e.g., diabetic diets, anti-reflux diet) or for reasons related to conscience (e.g., vegetarian diets). These diets typically resemble the meals provided to the rest of the population but are modified to accommodate the inmates’ dietary restrictions and beliefs. Typically, inmates’ diet prescriptions remain the same throughout incarceration. They usually request a religious diet or a diet of conscience at the beginning of their incarceration. The only exception is the medical diet since it is commonly prescribed during incarceration, because an inmate may get diagnosed with a new medical condition.

Our recent study found that 73% of inmates in Canada gained weight behind bars, putting them at increased risk of becoming obese [5]. We observed that obesity rates increased from 26% to 46% during incarceration [5]. Our findings also revealed that inmates behind bars gained more weight, at a faster rate, than adults in the general population in Canada. Given these findings, weight gain in prison is generally deemed as undesirable since most inmates go into prison with normal or overweight bodyweights, and become overweight or obese during incarceration [5]. It is generally known that food intake [6] and the food environment influence weight status [7]. However, we do not know how the observed weight gain in the inmate population was related to their reported food intake, and/or if the inmates who gained more weight were following the standardized menu, a special diet or their own meal plan (based on the feeding system of their institution).

A few studies, mostly out of the United States, have assessed inmates’ diets and food intake and have shown high variations in food provision between penitentiaries [8]. In most cases, menus served to inmates have been shown to be higher in sodium and sugar; and lower in fiber, magnesium, potassium, vitamin D and vitamin E than the daily recommended intake [8–10]. To our knowledge, there are no published studies on inmates’ food intake in Canada. To fill this knowledge gap, this study examined for the first time reported eating habits and diet prescriptions of inmates based on their body weight changes during incarceration in Canadian federal penitentiaries. In other words, the objective of this study was to examine the eating habits of inmates who gained the most weight in prison. Firstly, we hypothesized that inmates who gained the most weight would report having poorer diets (e.g. high in soda drinks, junk food, and pastries, and low in fruits and vegetables) compared with those who gained less weight. We also hypothesized that weight gain would be associated with frequent consumption of foods deemed unhealthy from the commissary store (or ‘canteen’). Lastly, we hypothesized that inmates who gained more weight would be following the standardized menu or their own meal plan (in small group meal plan), instead of an accommodation diet (medical, religious or diet of conscience).

## Materials and methods

This retrospective cohort study explored how weight gain in the inmate population was related to their reported food intake, and/or their meal plan (e.g., the standardized menu, their own meal plan or a special diet) in Canadian penitentiaries [11]. Participants for this research project were male and female inmates who volunteered to take part in the study. To participate, they had to be incarcerated for at least 6 months in their current federal institutions in the Ontario or Atlantic regions. Critically ill inmates admitted to the prison hospital and pregnant inmates were excluded from the study. In the Ontario region, we collected data from inmates housed in 5 institutions near Kingston (of the 7 institutions in the Ontario region) [12]. These institutions were selected for geographical feasibility reasons. In the Atlantic region, we collected data from inmates housed in all 5 institutions in New Brunswick and Nova Scotia [12]. We used a convenience sample, and advertised the study by offering information sessions with the inmate committee in each of the institutions where we were collecting data to encourage inmates to volunteer. In addition, there were advertisements about the study posted on the prison telecommunication service. Inmates were asked to submit their names to a designated staff member in the penitentiary.

### Recruitment strategy

In the beginning, we drew a list of random inmates, and called them down (over loud speaker) to our offices to ask if they wanted to participate. We had a very low response rate with this approach since inmates found it stressful to be called down without knowing why. With this approach, the vast majority of inmates refused to participate. However, the recruitment strategy described above where we asked for volunteers was more successful since the inmates were empowered to volunteer if they wanted to. Getting them to submit their names to a staff member also increased their confidence in the study. We did not keep track of who volunteered and who did not. For ethical reasons (i.e., confidentiality), we could not gather data on inmates who did not volunteer to participate since we did not have their consent to access their administrative files. At the time of data collection, there were approximately 3000 inmates living in the penitentiaries who participated in the study. From that population, approximately 1600 were eligible to participate. Overall, roughly 50% of eligible inmates volunteered to participate in our study. The prison setting is known to be challenging for recruiting participants because inmates are not typically interested in participating in this type of research study. Our challenge was getting inmates to volunteer and to wait while we coordinated with security to organize the interview. From our literature review, we found that the participation rates are generally quite low for studies on prisoners’ weight and weight change, where inmates are asked to participate in an interview and to have anthropometric measurements taken. For example, a French study only managed to recruit 18 male participants because of lack of interest in the study [13]. In addition, an American study had a sample of 103 participants [14], because recruiting inmates to participate in body weight related research is challenging. Typically, studies on weight during incarceration with large sample sizes were using secondary data.

### Data collection

Research assistants (who were trained registered dietitians) gathered data from 754 inmates who volunteered for a 30-minute face-to-face interview from May 2016 until September 2017. They also objectively measured participants’ height and weight following a standardized protocol, and subtracted current anthropometric data from those measured at the admission and recorded in the medical charts of participants to determine anthropometric changes during incarceration. The main outcome measures for this study were body weight change (kg), body mass index (BMI) change (kg/m^2^) and annual weight change (kg/year).

During the interview, we gathered self-reported data on food intake with a food frequency questionnaire. The questions were based on the Canadian Health Measures Survey (Cycle 3-household questionnaire: Statistics Canada-Cycle 3), with slight modifications to fit the prison setting and to make the questions easier for inmates to answer (based on feedback from inmates while piloting the questionnaire). The full food frequency questionnaire is presented in S1 Table in the supplementary material section of this article. We asked the following specific questions regarding food consumed from the canteen: “What type of food do you typically consume from canteen?*”* (response options: nothing, junk food (e.g., chocolate bars, chips, cakes etc.), healthy foods (e.g. yogurt, dried fruit, nuts, tuna, oats), beverages (specify type), supplements (specify type), or other (specify). Lastly, we gathered data on diets (medical, religious and diets of conscience) from their digital medical charts.

### Covariates

We adjusted our models using the following covariates: sex, age, ethnicity, region, feeding system and length of incarceration as they were defined by Correctional Service Canada’s standard and taken from inmates’ chart. For physical activity, we adjusted for the reported time (number of minutes) spent doing moderate to vigorous physical activity per day. For diet, we adjusted for reported vegetables (indicator for healthy eating), and for sugar-sweetened beverages (indicator for unhealthy eating) consumed daily.

### Statistical analysis

We performed chi-square and nonparametric median comparison tests (Wilcoxon and Kruskal-Wallis) to detect statistically significant changes in anthropometric measurements (weight change, BMI change, yearly weight change). We then assessed the weight outcomes with reported diet factors (special diet, canteen food and supplementation) and reported food intake (from food frequency questionnaire), to determine how the diet related factors differed depending on the amount of weight inmates gained. These tests were performed on the median because the data did not have a normal distribution (it was skewed to the right). In addition, we performed quantile regression analysis to examine whether associations were different for medium, and high percentiles by modelling the 0.5 (the median), 0.75 and 0.90 quantiles of the BMI change distribution adjusted by sex, ethnicity, region, length of incarceration, substance abuse, physical activity, diet and feeding system. We opted for the conditional quantile regression model [15–17] instead of the multivariate regression analysis on the mean, because the residuals (from the multiple regression model) did not meet the model assumptions (i.e., normality, linearity, homoscedasticity). Statistical analyses were performed using the Statistical Analysis Software (SAS) version 9.4. The level of statistical significance was set at p<0.05 for all analyses.

### Ethics approval

We obtained ethics approval through the Research Ethics Board at the University of Ottawa and the Research branch at Correctional Service Canada. Inmates volunteered to participate and provided their consent by signing our consent from. Since many inmates hesitate to sign documents or forms, because of low literacy and/or fear of reprisal, participants could provide verbal consent if they preferred [18]. All personal data collected were coded to ensure confidentiality.

## Results

Table 1 presents sociodemographic information by diet-related factors, such as feeding system, diet and purchases from the commissary store (or “canteen”). The vast majority of inmates (95%) reported purchasing food from the commissary store (or “canteen”). Age was associated with diet-related factors; younger inmates typically reported making healthier food choices from the commissary store (or “canteen”) than older inmates. We also observed ethnic variations, with Caucasian (40%) and Aboriginal (47%) inmates more likely to report not purchasing healthy foods from canteen compared to black (22%) and other ethnic minorities (25%). We observed regional variations in diet prescriptions. A higher proportion of inmates were on a special diet in the Ontario region (27%) compared to the Atlantic region (19%). Variation based on sex, language groups (Anglophone vs Francophone), and ethnic variations were associated with other diet-related factors (see Table 1).

**Table 1:** Sociodemographic information by diet-related factors (N = 754).

		All N (%)	Sex		p-value	Age %					p-value	Region %		p-value	Language		p-value	Ethnicity %				p-value
			Male	Fem		18≤24 years	≥25≤ 34 years	≥35≤44 years	≥45≤64 years	≥65 years		Atl.	Ont.		Anglo	Franco		Cau.	Black	Abori	Other	
All N (%)		754 (100)	672 (89)	82 (11)		63 (8)	221 (29)	176 (23)	249 (33)	45 (6)		311 (41)	443 (59)		645 (86)	109 (14)		470 (62)	124 (16)	106 (14)	54 (7)	
Feeding system	Tray	195 (26)	28	13	<0.0001*	41	35	26	16	11	<0.0001*	15	33	<0.0001*	27	19	0.0020*	19	34	39	44	<0.0001*
	Cafeteria	225 (30)	33	0		33	32	28	30	20		35	26		27	44		31	31	24	24	
	Meal plan	334 (44)	39	87		25	33	46	53	69		49	41		46	37		50	35	38	31	
Special diet	Yes	180 (24)	24	21	0.4797	33	33	21	16	24	<0.0001*	19	27	0.0134*	24	22	0.6234	14	57	17	46	<0.0001*
	No	574 (76)	76	79		67	67	79	85	76		81	73		76	78		86	43	83	54	
Diet type	Medical	55 (31)	28	59	0.0165*	14	18	35	54	55	<0.0001*	40	26	0.0066*	27	54	0.0677	56	6	56	16	<0.0001*
	Religious	75 (42)	45	6		62	51	43	16	18		43	41		44	25		20	62	28	52	
	Diet of conscience	50 (28)	27	35		24	31	22	30	27		17	33		29	21		24	32	17	32	
Canteen purchase	Yes	718 (95)	95	96	0.7879	98	97	92	95	98	0.1194	96	95	0.5212	95	94	0.6991	96	97	92	94	0.4547
	No	36 (5)	5	4		2	3	8	5	2		4	5		5	6		4	3	8	6	
Canteen healthy food (N = 718)	Yes	452 (63)	65	49	0.0254*	74	70	64	56	50	0.0019*	58	66	0.0589	62	67	0.6112	60	78	53	75	0.0005*
	No	266 (37)	35	51		26	30	36	44	50		42	34		38	33		40	22	47	25	
Canteen junk food (N = 718)	Yes	528 (74)	72	86	0.0236*	81	72	73	74	68	0.2791	77	71	0.2555	73	77	0.6828	75	72	77	63	0.3627
	No	190 (26)	28	14		19	28	27	26	32		23	29		27	23		25	28	23	37	

A Wilcoxon test was used in analyses with two categories (special diet, canteen purchase, canteen-healthy food, canteen-junk food), and a Kruskal-Wallis test was used in analyses with three or more categories (feeding system and diet type). A p-value <0.05 was considered statistically significant. Fem: Female; Atl.: Atlantic; Ont.: Ontario; Anglo: Anglophone; Franc: Francophone; Cau: Caucasian; Abori: Aboriginal.

Table 2 presents sociodemographic information by food intake (as estimated with the food frequency questionnaire). Overall food frequency intake was similar regardless of sociodemographic factors, but some variations were seen by sex, region, language and ethnicity. For example, women reported drinking more fruit drinks; whereas men reported drinking more diet soft drinks. Younger inmates (≤ 64 years) drank regular soft drink more frequently; whereas older inmates (≥65 years) drank diet soft drinks more frequently. In addition, we observed regional and ethnic variations with regards to soft drink consumption and vegetable intake.

**Table 2:** Sociodemographic information by food frequency intake (N = 742).

	Daily intake	All (%)	Sex %		p-value	Age %					p-value	Region %			Language %		p-value	Ethnicity %				p-value
			Male	Fem		18≤24 years	≥25≤34 years	≥35≤44 years	≥45≤64 years	≥65 years		Atl.	Ont.		Anglo	Franc		Cau.	Black	Aboriginal	Other	
All N (%)		742 (100)	660 (89)	82 (11)		63 (8)	216 (29)	174 (23)	247 (33)	42 (6)		311 (42)	431 (58)		634 (85)	108 (15)		466 (63)	124 (17)	99 (13)	53 (7)	
**Vegetables** **per day**	0	24 (3)	3	2	0.0541	0	2	3	6	0	0.0544	2	6	<0.0001*	3	4	0.8175	3	3	6	4	0.0715
	<1	199 (27)	27	22		25	29	24	25	40		27	26		26	31		24	39	23	28	
	≥1<2	229 (31)	32	26		33	29	34	31	24		37	26		31	31		32	23	34	36	
	≥2<3	194 (26)	26	27		35	29	24	23	29		27	25		26	25		29	25	20	19	
	>3	96 (13)	12	23		6	11	15	16	7		6	18		13	10		13	10	16	13	
**Fruits per day**	0	32 (4)	4	4	0.0528	3	1	5	6	7	0.0688	3	5	0.1027	4	8	0.0076*	4	4	7	0	0.1346
	<1	159 (21)	22	21		17	21	21	21	31		20	23		20	31		20	31	17	21	
	≥1<2	223 (30)	30	34		33	28	32	32	17		35	27		31	24		31	24	29	36	
	≥2<3	161 (22)	21	30		27	23	25	18	17		20	23		23	15		22	17	23	28	
	>3	167 (23)	24	11		19	27	17	23	29		23	23		23	22		23	23	23	15	
**Fruit drinks per day**	0	558 (75)	78	51	<0.0001*	75	78	76	74	62	0.2504	77	74	0.3674	74	81	0.0134*	74	73	82	75	0.2094
	<1	104 (14)	12	33		17	14	14	13	17		12	16		14	17		14	19	8	19	
	≥1	80 (11)	10	16		8	8	10	13	21		11	11		12	3		12	9	10	6	
**Regular soft drinks per day**	0	314 (42)	43	38	0.6343	29	39	40	47	62	0.0046*	37	46	0.0008*	43	41	0.8800	45	30	48	38	0.0130*
	<1	327 (44)	43	49		52	51	45	38	26		52	38		44	46		42	52	37	57	
	≥1	101 (14)	14	13		19	10	14	15	12		11	16		14	13		13	19	14	6	
**Diet soft drinks per day**	0	649 (87)	86	98	0.0129*	98	90	90	83	76	0.0015*	89	86	0.5277	88	85	0.1810	86	90	89	89	0.4604
	<1	72 (10)	11	2		2	9	7	13	12		8	11		10	9		11	7	8	11	
	≥1	21 (3)	3	0		0	1	3	4	12		3	3		2	6		3	2	3	0	

A Kruskal-Wallis test was used in all analyses. A p-value <0.05 was considered statistically significant. Fem: Female; Atl.: Atlantic; Ont.: Ontario; Anglo: Anglophone; Franc: Francophone; Cau: Caucasian.

Table 3 shows data on weight change during incarceration in relation to food systems, type of diet, quality of food and intake of supplements. In comparison to inmates without a special diet (who gained 5.8 kg), inmates on a medical diet gained more weight (7.5 kg). Whereas, inmates on a religious accommodation diet gained less weight (4.5 kg) and inmates on a diet of conscience lost weight (-0.3 kg). The same patterns were seen in BMI and annual weight change outcomes. Furthermore, inmates who gained significantly less weight also reported eating healthy foods and protein supplements from the commissary store (or “canteen”). The same patterns were observed with BMI change.

**Table 3:** Change in median weight, body mass index (BMI) and annual weight between admission and study follow-up in relation to feeding systems, type of diet, quality of food and intake supplements.

		All N (%)	Median weight change kg (95% CI)	p-value	Median BMI change kg/m^2^ (95% CI)	p-value	Annual weight change kg/year (95% CI)	p-value
**All**		754 (100)	+5.6 (4.8–6.4)		+1.8 (1.5–2.1)		+1.4 (1.0–1.8)	
**Feeding system**	Tray	195 (26)	+ 5.1 (3.5–6.6)	0.5515	+ 1.6 (1.2–2.1)	0.4641	+ 1.5 (0.9–2.1)	0.5602
	Cafeteria	225 (30)	+ 5.0 (3.4–6.6)		+ 1.7 (1.2–2.2)		+ 1.3 (0.7–1.9)	
	Meal plan	334 (44)	+ 6.9 (5.6–8.2)		+ 2.2 (1.8–2.2)		+1.3 (0.6–2.1)	
**Type of diet at interview**	No diet	586 (78)	+ 5.8 (4.8–6.8)	0.0002*	+ 1.9 (1.6–2.2)	0.0060*	+ 1.5 (1.0–2.0)	0.0210*
	Medical	98 (13)	+ 7.5 (4.6–10.4)		+ 2.4 (1.4–3.4)		+ 1.3 (0.2–2.4)	
	Religious	41 (5)	+ 4.5 (1.4–7.6)		+ 1.5 (0.5–2.5)		+ 1.1 (0.2–2.0)	
	Diet of conscience	29 (4)	-0.3 (-3.8–3.2)		-0.1 (-1.2–1.0)		-0.1 (-0.8–0.6)	
**Canteen purchase**	Yes	718 (95)	+5.5 (4.6–6.3)	0.2932	+ 1.8 (1.5–2.1)	0.2912	+ 1.4 (1.0–1.8)	0.7874
	No	36 (5)	+7.0 (3.3–10.7)		+ 2.3 (1.1–3.4)		+ 1.1 (-1.0–3.2)	
**Healthy food from canteen (N = 718)**	Yes	452 (63)	+ 4.8 (3.7–5.7)	0.0055*	+ 1.6 (1.5–2.3)	0.0036*	+ 1.2 (0.8–1.6)	0.0363*
	No	266 (37)	+ 8.0 (6.4–9.6)		+ 2.5 (2.4–3.4)		+ 1.8 (0.9–2.7)	
**Junk food from canteen (N = 718)**	Yes	528 (74)	+ 5.6 (4.5–6.6)	0.3651	+ 1.9 (1.5–2.2)	0.3245	+ 1.6 (1.1–2.0)	0.3433
	No	190 (26)	+ 5.1 (3.4–6.7)		+ 1.6 (1.1–2.1)		+ 1.0 (0.3–1.8)	
**Protein supplement from canteen (N = 718)**	Yes	99 (14)	+ 3.6 (1.7–5.5)	0.0160*	+ 1.2 (0.6–1.8)	0.0098*	+ 0.9 (-0.3–2.1)	0.1704
	No	619 (86)	+6.0 (4.8–7.2)		+ 1.9 (1.6–2.2)		+ 1.5 (1.1–1.9)	
**Vitamin supplement from canteen (N = 718)**	Yes	63 (9)	+ 4.4 (1.2–7.6)	0.3470	+ 1.5 (0.3–2.7)	0.4334	+ 0.5 (-0.5–1.5)	0.1264
	No	655 (91)	+ 5.6 (4.7–6.5)		+1.8 (1.5–2.1)		+1.5 (1.1–1.9)	

A Wilcoxon test was used in analyses with two categories (Canteen purchase, Healthy food, Junk food, Protein supplement, Vitamin supplement), and a Kruskal-Wallis test was used in analyses with three or more categories (Feeding system and Type of diet).

*p-value <0.05 was considered statistically significant.

The average length between admission and follow-up was 5.0 ± 8.3 years. CI = confidence interval.

Table 4 and Table 5 present data on changes in median weight, median BMI and median annual weight for inmates in relation to the frequency of different food group consumption. Inmates who gained the most weight (15.7 kg) reported not eating vegetables, whereas inmates who gained less weight (4.8 kg) reported consuming vegetables more than 3 times per day. Similar patterns were observed in inmates who reported eating healthy food regularly, such as fruit, cereal, dairy and legumes. Moreover, inmates who gained more weight also reported eating high calorie non-nutritious foods regularly (i.e. pastries and regular soft drinks). Similar patterns of associations were observed for BMI and annual weight change. Data on other foods (bread, rice, poultry, fish, red meat, eggs, peanut butter, nuts, chocolate, ice cream and water) where not found to be significantly different for inmates based on their weight gain during incarceration.

**Table 4:** Change in median weight, body mass index (BMI) and annual weight between admission and study follow-up in relation to food group intake (food frequency questionnaire).

	**Daily intake**	**All N (%)**	**Median weight change kg (95% CI)**	**p-value**	**Median BMI change kg/m^2^ (95% CI)**	**p-value**	**Annual weight change kg/year (95% CI)**	**p-value**
**All**		742 (100)	+ 5.6 (4.8–6.4)		+ 1.8 (1.5–2.1)		+1.4 (1.0–1.8)	
**Vegetables per day**	0	24 (3)	+15.7 (8.8–22.5)	0.0043*	+ 4.9 (2.7–7.1)	0.0037*	+ 4.6 (1.9–7.2)	0.0453*
	<1	199 (27)	+ 6.0 (4.3–7.7)		+ 2.0 (1.5–2.5)		+ 1.5 (0.7–2.3)	
	≥1<2	223 (30)	+ 4.8 (3.3–6.3)		+ 1.7 (1.2–2.2)		+ 1.5 (0.7–2.2)	
	≥2<3	194 (26)	+ 6.1 (4.3–7.9)		+ 1.9 (1.3–2.4)		+ 1.2 (0.5–1.9)	
	>3	96 (13)	+ 4.8 (3.3–6.3)		+ 1.6 (0.9–2.2)		+ 1.0 (0.3–1.8)	
**Fruits per day**	0	32 (4)	+ 14.3 (10–18.5)	0.0030*	+ 4.7 (3.1–6.3)	0.0010*	+ 3.0 (0.3–5.7)	0.0186*
	<1	159 (21)	+ 7.5 (5.6–9.4)		+ 2.4 (1.8–3.0)		+ 1.9 (0.8–3.0)	
	≥1<2	223 (30)	+ 5.2 (3.6–6.8)		+ 1.8 (1.3–2.3)		+ 1.3 (0.5–2.0)	
	≥2<3	161 (22)	+ 5.0 (3.3–6.7)		+ 1.6 (1.1–2.1)		+ 1.4 (0.6–2.2)	
	>3	167 (23)	+ 4.0 (2.2–5.8)		+ 1.3 (0.8–1.8)		+ 0.9 (0.4–1.5)	
**Bread per day**	0	88 (12)	+ 7.5 (5.3–9.7)	0.1127	+ 2.5 (1.7–3.2)	0.1383	+ 1.8 (0.4–3.3)	0.4474
	<1	203 (27)	+ 4.5 (3.0–6.0)		+ 1.4 (0.9–1.9)		+ 1.4 (0.6–2.1)	
	≥1	451 (61)	+6.0 (4.9–7.1)		+ 2.0 (1.6–2.4)		+ 1.4 (0.9–1.9)	
**Cereal per day**	0	202 (27)	+ 7.6 (5.9–9.3)	0.0264*	+ 2.5 (1.9–3.0)	0.0246*	+ 2.2 (1.4–3.0)	0.1880
	<1	272 (37)	+ 5.0 (3.5–6.5)		+ 1.7 (1.2–2.2)		+ 1.1 (0.5–1.8)	
	≥1	268 (36)	+ 4.9 (3.6–6.2)		+ 1.6 (1.2–2.0)		+ 1.3 (0.8–1.8)	
**Rice and pasta per day**	0	51 (7)	+ 7.5 (4.3–10.7)	0.5374	+ 2.4 (1.2–3.6)	0.4627	+ 2.3 (0.0–4.6)	0.2449
	<1	554 (75)	+ 5.4 (4.3–6.4)		+ 1.8 (1.4–2.1)		+ 1.2 (0.8–1.7)	
	≥1	137 (18)	+ 5.5 (3.8–7.2)		+ 1.8 (1.3–2.3)		+ 1.8 (0.8–2.8)	
**Dairy per day**	0	316 (43)	+7.5 (6.2–8.7)	0.0141*	+ 2.3 (1.9–2.7)	0.0186*	+ 1.7 (1.2–2.3)	0.1879
	<1	119 (16)	+6.0 (3.6–8.4)		+ 2.0 (1.2–2.8)		+ 1.1 (-0.2–2.5)	
	≥1<2	129 (17)	+3.2 (1.1–5.3)		+ 0.9 (0.3–1.5)		+ 0.9 (0.1–1.7)	
	≥2	178 (24)	+5.1 (3.3–6.8)		+ 1.6 (1.0–2.2)		+ 1.4 (0.7–2.1)	
**Poultry per day**	0	92 (12)	+ 4.5 (1.5–7.5)	0.3078	+ 1.6 (0.6–2.5)	0.2552	+ 1.0 (-0.1–2.1)	0.5046
	<0.5	327 (44)	+ 7.1 (5.8–8.4)		+ 2.4 (2.0–2.8)		+ 1.4 (0.9–2.1)	
	≥0.5	323 (44)	+ 4.8 (3.6–6.0)		+ 1.6 (1.2–2.0)		+ 1.4 (1.0–2.0)	
**Fish per day**	0	178 (24)	+7.1 (5.0–9.1)	0.4966	+ 2.3 (1.6–2.9)	0.4575	+ 1.7 (0.9–2.5)	0.8412
	<0.5	488 (66)	+ 5.2 (4.1–6.2)		+ 1.7 (1.4–2.0)		+ 1.4 (0.9–1.8)	
	≥0.5	76 (10)	+ 5.2 (3.1–7.2)		+ 1.8 (1.1–2.4)		+ 1.6 (0.4–2.8)	
**Red meat per day**	0	89 (12)	+ 4.5 (1.9–7.1)	0.3538	+ 1.6 (0.7–2.5)	0.4220	+ 1.3 (0.2–2.3)	0.6425
	<0.5	417 (56)	+ 6.5 (5.4–7.6)		+ 2.1 (1.7–2.5)		+ 1.5 (1.0–2.1)	
	≥0.5	236 (32)	+ 4.9 (3.4–6.4)		+ 1.6 (1.1–2.1)		+ 1.1 (0.5–1.7)	
**Eggs per day**	0	104 (14)	+6.7 (4.4–9.0)	0.5201	+ 2.2 (1.4–2.9)	0.4856	+ 1.5 (0.0–3.0)	0.4576
	<0.5	331 (45)	+ 5.5 (4.2–6.8)		+ 1.8 (1.4–2.2)		+ 1.3 (0.8–1.8)	
	≥0.5	307 (41)	+ 5.5 (4.1–6.9)		+ 1.7 (1.3–2.1)		+ 1.5 (1.0–2.1)	
**Peanut butter**	0	135 (18)	+ 7.5 (5.7–9.3)	0.5116	+ 2.4 (1.8–3.0)	0.4779	+1.8 (0.7–2.9)	0.2909
	<0.5	242 (33)	+ 5.7 (4.2–7.2)		+1.9 (1.4–2.4)		+ 1.4 (0.7–2.1)	
	≥0.5	365 (49)	+ 5.0 (3.7–6.3)		+ 1.6 (1.2–2.0)		+ 1.3 (0.8–1.8)	

A Kruskal-Wallis test was used in all analyse.

*p-value <0.05 was considered statistically significant.

The average length between admission and follow-up was 5.0 ± 8.3 years. CI = confidence interval.

**Table 5:** Change in median weight, body mass index (BMI) and annual weight between admission and study follow-up in relation to food group intake (food frequency questionnaire).

	Daily intake	All N (%)	Median weight change kg (95% CI)	p-value	Median BMI change kg/m^2^ (95% CI)	p-value	Annual weight change kg/year (95% CI)	p-value
**Nuts and seeds per day**	0	455 (61)	+ 6.0 (4.9–7.1)	0.1550	+ 1.9 (1.5–2.3)	0.4027	+ 1.6 (1.0–2.2)	0.0599
	<0.5	179 (24)	+ 5.6 (3.9–7.3)		+ 2.0 (1.4–2.6)		+ 1.5 (0.9–2.0)	
	≥0.5	108 (15)	+ 4.3 (2.2–6.3)		+ 1.6 (0.9–2.2)		+ 0.7 (-0.1–1.5)	
**Legumes per day**	0	235 (32)	+ 6.5 (4.8–8.2)	0.0110*	+ 2.2 (1.6–2.8)	0.0096*	+ 1.5 (0.6–2.5)	0.0552
	<0.5	417 (56)	+ 6.0 (4.9–7.1)		+ 1.9 (1.5–2.2)		+ 1.5 (1.0–1.9)	
	≥0.5	90 (12)	+ 4.0 (2.1–5.9)		+ 1.3 (0.7–1.9)		+ 0.8 (0.0–1.7)	
**Pastries (cakes and cookies) per day**	0	140 (19)	+ 5.5 (3.6–7.4)	0.0246*	+ 1.7 (1.1–2.3)	0.0137*	+ 1.3 (0.6–2.0)	0.0091*
	<0.29	294 (40)	+ 5.1 (3.8–6.3)		+ 1.7 (1.2–2.1)		+ 1.6 (1.0–2.2)	
	≥0.29<0.5	136 (18)	+ 4.3 (2.4–6.2)		+ 1.4 (0.8–2.0)		+ 0.8 (0.1–1.4)	
	≥0.5	172 (23)	+ 8.3 (6.4–10.2)		+ 2.8 (2.2–3.4)		+ 1.8 (0.5–3.2)	
**Chocolate per day**	0	417 (56)	+ 5.9 (4.8–7.0)	0.5722	+ 1.9 (1.5–2.3)	0.6211	+ 1.4 (1.0–1.9)	0.9569
	<0.29	219 (30)	+ 5.0 (3.4–6.6)		+ 1.7 (1.2–2.2)		+ 1.3 (0.4–2.1)	
	≥0.29<0.5	58 (8)	+ 6.1 (2.9–9.3)		+ 2.1 (1.0–3.2)		+ 1.8 (0.1–3.5)	
	≥0.5	48 (6)	+ 5.8 (2.8–8.7)		+ 1.8 (0.9–2.7)		+ 1.2 (0.4–2.1)	
**Ice cream per day**	0	371 (50)	+ 5.5 (4.3–6.7)	0.7982	+ 1.8 (1.4–2.2)	0.8413	+ 1.4 (0.8–2.0)	0.8547
	<0.29	238 (32)	+ 5.9 (4.3–7.5)		+ 1.8 (1.3–2.3)		+ 1.3 (0.6–2.0)	
	≥0.29<0.5	62 (8)	+ 7.5 (4.6–10.4)		+ 2.4 (1.6–3.2)		+ 1.5 (0.3–2.6)	
	≥0.5	71 (10)	+ 5.0 (2.6–7.4)		+ 1.7 (0.9–2.5)		+ 1.6 (0.1–3.0)	
**Regular soft drinks per day**	0	314 (42)	+5.3 (4.0–6.5)	0.0065*	+ 1.7 (1.3–2.1)	0.0071*	+ 1.3 (0.7–2.0)	0.0076*
	<1	327 (44)	+4.8 (3.5–6.1)		+ 1.7 (1.3–2.1)		+ 1.2 (0.6–1.7)	
	≥1	101 (14)	+10 (7.7–12.3)		+ 3.2 (2.4–4.0)		+ 3.3 (2.2–4.3)	
**Diet soft drinks per day**	0	649 (87)	+ 5.5 (4.6–6.4)	0.3489	+ 1.8 (1.5–2.1)	0.3111	+1.5 (1.1–1.9)	0.5529
	<1	72 (10)	+7.3 (3.7–10.8)		+ 2.2 (1.1–3.3)		+ 0.9 (-0.7–2.5)	
	≥1	21 (3)	+ 12 (1.9–22.1)		+ 4.1 (1.0–7.2)		+ 1.0 (-0.5–2.4)	
**Fruit drinks per day**	0	558 (75)	+ 5.3 (4.3–6.2)	0.5682	+ 1.8 (1.5–2.1)	0.4747	+ 1.4 (1.0–1.8)	0.3306
	<1	104 (14)	+ 7.0 (4.5–9.4)		+ 2.3 (1.5–3.1)		+ 1.4 (0.0–2.9)	
	≥1	80 (11)	+ 8.5 (5.6–11.4)		+ 3.0 (2.0–3.9)		+ 1.3 (-0.6–3.3)	
**Water per day**	0	26 (4)	+10.1 (5.9–14.1)	0.0916	+ 3.4 (2.1–4.7)	0.0741	+ 1.8 (-0.7–4.3)	0.1391
	<3	92 (12)	+7.2 (4.7–9.6)		+ 2.5 (1.6–3.2)		+ 2.9 (1.4–4.4)	
	≥3<7	205 (28)	+5.0 (3.4–6.6)		+ 1.6 (1.1–2.1)		+ 1.4 (0.6–2.2)	
	≥7<10	156 (21)	+4.9 (3.2–6.5)		+ 1.6 (1.0–2.1)		+ 1.0 (0.5–1.6)	
	≥10	263 (35)	+6.1 (4.6–7.6)		+ 1.9 (add)		+ 1.4 (0.8–2.1)	

A Kruskal-Wallis test was used in all analyse.

*p-value <0.05 was considered statistically significant.

The average length between admission and follow-up was 5.0 ± 8.3 years. CI = confidence interval.

Table 6 presents the results of a quantile regression coefficients analysis that confirms the association between BMI change and diet. For inmates from the groups with the highest weight change (50^th^, 75^th^ and 90^th^ percentiles), inmates who reported eating no vegetables had respectively 2.3, 3.4 and 0.9 point of BMI higher than inmates who reported eating vegetables daily. Moreover, BMI gain was significantly higher for inmates above the age of 65, for inmates of Aboriginal decent and inmates who were incarcerated the longest (length of incarceration >5 years). These findings were adjusted for socio-demographic factors (sex, age, region, language and ethnicity) as well as for other factors (length of incarceration, physical activity, substance abuse and feeding system).

**Table 6:** Quantile regression coefficients analysis for estimated BMI change based on the daily frequency of vegetable intake and sociodemographic factors and length of incarceration.

Variables		50^th^ percentile (CI 95%)	75^th^ percentile (CI 95%)	90^th^ percentile (CI 95%)
**Diet- vegetable intake per day**	0	+2.3 (-1.19, 4.83)	+3.4† (2.04, 4.25)	+0.9† (0.15, infinity)
	<1	+0.01 (-0.64, 0.84)	+0.46 (-0.72, 1.39)	-0.3 (-2.01, 1.61)
	≥1<2	-0.14 (-0.96, 0.61)	-0.09 (-1.13, 0.59)	-0.88 (-2.59, 0.70)
	≥2<3	0 (reference)	0 (reference)	0 (reference)
	≥3	-0. 83 (-1.51, 0.35)	-0.71 (-1.74, 0.19)	-2.55 (-4.28, 0.53)
**Sex**	Male	0 (reference)	0 (reference)	0 (reference)
	Female	-0.14 (-0.68, 0.58)	+1.44 (-0.80, 3.05)	+2.88† (1.29, 7.14)
**Age**	18≤24 years	-2.39 (-4.16, 0.22)	-2.41† (-5.91, -0.69)	-2.38 (-5.47, 0.97)
	≥25≤34 years	-1.89 (-3.36, 0.40)	-2.2† (-5.06, -0.44)	-2.93† (-5.58, -0.32)
	≥35≤44 years	-1.7 (-3.22, 0.77)	-2.31† (-5.31, -0.40)	-1.6 (-4.49, 0.96)
	≥45≤64 years	-1.04 (-2.50, 1.56)	-0.79 (-3.43, 0.66)	-0.9 (-3.27, 1.40)
	≥65 years	0 (reference)	0 (reference)	0 (reference)
**Ethnicity**	Caucasian	0 (reference)	0 (reference)	0 (reference)
	Black	+0.36 (-0.34, 0.96)	-0.04 (-0.90, 1.26)	-0.15 (-1.78, 2.11)
	Aboriginal	+1.21 (-0.50, 2.06)	+1.32† (0.57, 2.32)	+0.88 (-0.56, 2.54)
	Other	-0.46 (-1.28, 0.43)	-1.07† (-1.54, -0.03)	-1.98 (-3.19, 0.69)
**Length of incarceration**	≤18 months	0 (reference)	0 (reference)	0 (reference)
	>18 m ≤5 y	-0.34 (-0.92, 0.32)	-0.22 (-0.70, 0.46)	-0.28 (-1.52, 0.55)
	>5 years	+0.64 (-0.26, 1.64)	+1.57† (0.56, 2.60)	+1.93† (0.70, 3.68)

† <0.05 was considered statistically significant.

The results presented were adjusted for the factors seen in the table and other factors (language, feeding system, physical activity and substance abuse) (data not shown in table). BMI, body mass index; CI, confidence interval. The vegetable daily intake represents the reported frequency of vegetable consumption (0 means the inmate reported never eating vegetables, <1 means the inmate reported eating vegetables less than once daily, ≥1<2 means the inmate reported eating vegetables once or twice per day etc.)

## Discussion

Our findings show that the inmates who gained the most weight during incarceration reported infrequent intake of healthy foods (i.e. vegetables, fruit, cereal, dairy and legumes) and more frequent intake of unhealthy foods (i.e. pastries and regular soft drinks). For example, inmates with the highest weight increase (15.7 kg) reported never eating vegetables while inmates who gained less weight (4.8 kg) reported eating vegetables at least 3 times daily. Inversely, the weight gain was lower for inmates who limited their intake of non-nutritious high calorie foods. This was true for the food coming from food services through institutional meals, and for the food purchased from the commissary store (or “canteen”). These observations are well supported in nutrition research, where low calorie, high fiber foods (such as fruits and vegetables) are associated with lower body weight and lower BMI [6, 19, 20], whereas non-nutritious energy dense foods are associated with obesity and higher BMI [6, 21].

We observed that inmates who gained the least during incarceration also reported consuming healthy foods from the commissary store (or “canteen”). As such, we observed a median weight gain of 0.48 kg in inmates who reported purchasing healthy foods from the commissary store (or “canteen”) (e.g. legumes, tuna fish, oatmeal and nuts). Whereas, inmates who gained a median of 8 kg, reported not consuming these healthy foods. In addition, inmates who only gained a median 3.6 kg reported consuming protein supplements, compared to a median 6 kg weight gain for inmates who reported not taking protein supplements. Moreover, the consumption of commissary (or “canteen”) foods appears to influence inmates’ weight gain more than the food service system of the institution, since the data on commissary store (or “canteen”) use was associated with weight gain, whereas the food system of the penitentiary was not. This could in part explain why penitentiaries were deemed to be obesogenic since there are commissary stores (or “canteens”) in all institutions (regardless of the feeding system used in the penitentiary).

The food purchased at the commissary stores (or “canteen”) appeared to influence weight change outcomes more than the feeding system of the penitentiaries. That means targeting canteens and their food availability will likely be an effective tool when planning weight management interventions in prison. This is interesting since food available for purchase at canteens may vary between institutions, whereas the food provided by food service is controlled (by the standardized menu and related policies). As previously mentioned, food offerings for central feeding systems (with tray delivery and cafeteria) follow the same standardized menu across Canada. In other words, for both regions where data was collected the food available was the same (the menu or the grocery list for small group meal plan). This is interesting since weight gain during incarceration did not significantly differ based on the food system. That means, it did not appear to make a difference whether inmates were being fed in the highly structured food environment such as the central production and tray delivery system in comparison to the unstructured nature of the small group meal plan. With the tray delivery system, inmates get very little say in what and when they eat. The food is controlled by Correctional Service Canada and follows the standardized meal plan (based on Canada’s Food Guide and provides ~2600 kcal daily for male inmates, and ~2000 kcal for female inmates), and delivered to them directly in their cells. In this setting, inmates eat alone and have little opportunity to trade food with others. In the second food system (cafeteria), the food production is also centralized and follows the same standardized menu, but there is more trading between inmates since they all eat together in the dining area. Lastly, in the small group meal plan program there is a large potential for variability in meal plans since it is left to the discretion of each inmate to decide what food to purchase within their allotted food budget of ~$35 per week. With this feeding system, inmates get to choose what food to purchase from a standardized list that is the same across Canada. Our findings suggest that the feeding system did not significantly influence weight gain during incarceration.

Although weight gain was not associated with the feeding system, our findings suggest that the menu served to inmates was influential to weight gain since inmates on special diets had different weight gain patterns. Inmates with a median weight gain of 5.8 kg were on the regular meal plan (or not following a special diet). Whereas, inmates with less weight gain (4.5 kg) were following a religious diet; and some inmates who lost weight (-0.3 kg) were following a diet of conscience (usually a vegetarian meal plan). This suggests that the meal plans for religious accommodations and diets of conscience may provide different nutritional content. However, it is not known at this time how these modified diets compare to the standardized regular menu in terms of calories and other nutrients. The finding that inmates who gained the most (7.5 kg) were on medical diets was unexpected and concerning. It is possible that the reduced calories from the medical diets could cause inmates to seek extra calories elsewhere (from the commissary store or “canteen”) and consequently lead to weight gain. It is also possible that inmates who gain more weight during incarceration were then subsequently placed on a medical diet. In the second scenario, the medical diets were not necessarily contributing to weight gain, but were used as a tool to manage existing weight gain. Lastly, it is possible that the weight gain observed in inmates on a medical diet was related to medication use, since it is well established that some medications may lead to weight changes [22, 23]. At this point, we do not know why inmates on a medical diet gain more weight than other inmates, but it would be worth exploring in future research projects.

There are reports of inmates using junk food (chips, soft drinks and chocolate bars) from the commissary store (or “canteen”) as currency since the tobacco ban in 2008 [24]. Before the ban, tobacco and cigarettes were the main source of currency, but now inmates use junk food to exchange services and to gamble [24, 25]. This new phenomenon, where junk food is being used as a currency, may explain the obesogenic effect of the commissary store (or “canteen”). Junk food has now become omnipresent, and possibly consumed more frequently by all inmates and staff. There is data to support this peculiarity, but it is well documented that food is a big part of the prison micro-economy and the black market [1]. A more in-depth examination of food as currency would be worth exploring in future research.

Lately, the food environment has been identified as a key determinant for healthy eating and priority area for research. Many governments are developing public policies in an attempt to improve the food environment and improve population health [26]. Across Canada, we have seen multiple examples of successful initiatives, such as guideline for healthy food environments in British Columbia schools [27], the Ontario government offered workshops to transform the food environment by focusing on capacity building [28] and measuring the food environment by Health Canada [26]. These innovative approaches coincide well with this research project since the food environment in penitentiaries are “closed environments” that are heavily controlled by public policy and regulations. Therefore, allowing an examination of how prison regulation contributes to food choices and subsequent weight changes. Thus far, there is little evidence that prison authorities are using a population based approach for improving the food environment in Canadian penitentiaries; since there are no publications on initiatives aimed at improving the food environment in the prison setting.

### Limitations

This study should be interpreted in light of the following limitations. First, the observational nature of the data precludes inferences about causality. Second, the data collected on food intake were self-reported by participants, and therefore subject to recall bias [29]. Furthermore, the data related to food was only collected once, which means we only have one time-point of the behavior of interest. Also, only the food frequency was reported and no information was available on the amount consumed (kcal), so it was not possible to estimate energy and nutrient intake. Finally, residual confounding by unmeasured variables is always a possibility in observational studies.

## Conclusion

In conclusion, inmates are vulnerable to weight gain while incarcerated in Canadian penitentiaries probably in part because of the food they eat (or do not eat). Inmates who gained the most weight reported lower healthy food consumption (vegetables, fruit, legumes, cereal, dairy and legumes). Inversely, inmates who gained less weight reported eating those healthy foods daily. Given food intake is a modifiable risk factor for weight gain during incarceration, the findings from our study could help decision makers when choosing how to feed inmates, and which foods to have available to them. The commissary store (or “canteen”) appears to be more influential on weight gain than the food provided by food services (run by Correctional Service Canada). Making the commissary (or “canteen”) an efficient target for weight loss interventions or a means to improve the food environment in prison. Further research could examine the effectiveness of interventions aimed at increasing healthy food consumption through the commissary store (or “canteen”). An analysis of existing interventions in the community could be helpful to see what could be adapted to the prison environment. Furthermore, an in-depth analysis of the modified diets provided to inmates with special dietary needs and beliefs could also provide more insight into weight gain during incarceration.

## Supporting information

S1 Table Food frequency questionnaire: How often do you eat these foods? (DOCX)
