# Supplementary material for: An exploration of reported food intake among inmates who gained body weight during incarceration in Canadian federal penitentiaries
Source: PLoS One. 2018 Dec 18;13(12):e0208768. doi: 10.1371/journal.pone.0208768 (PMC6298656; doi:10.1371/journal.pone.0208768)
Supplement: S1 Table — (DOCX) [file pone.0208768.s001.docx]

## Supporting Information

| Food | Per day | Per week | Per month | Never | Don’t know | Refusal |
| --- | --- | --- | --- | --- | --- | --- |
| 13a. Water |  |  |  |  |  |  |
| 13b. Skim milk (powder) |  |  |  |  |  |  |
| 13c. 2% or lactose free milk |  |  |  |   |  |  |
| 13d. Soy beverage |  |  |  |  |  |  |
| 13e. Regular pop |  |  |  |  |  |  |
| 13f. Diet pop |  |  |  |  |  |  |
| 13g. Sports drinks |  |  |  |  |  |  |
| 13h. Pure fruit juice |  |  |  |  |  |  |
| 13i.Fruit drinks or cocktail |  |  |  |  |  |  |
| 13j. Fruits |  |  |  |  |  |  |
| 13k. Vegetables (other than potatoes) |  |  |  |  |  |  |
| 13l. Potatoes |  |  |  |  |  |  |
| 13m.Poultry |  |  |  |  |  |  |
| 13n.Red meat (beef, pork) |  |  |  |  |  |  |
| 13o. Eggs |  |  |  |  |  |  |
| 13p. Peanut butter |  |  |  |  |  |  |
| 13q. Fish |  |  |  |  |  |  |
| 13r. Legumes (beans, tofu, lentils) |  |  |  |  |  |  |
| 13s. Bread |  |  |  |  |  |  |
| 13t. Cereal |  |  |  |  |  |  |
| 13u. Pasta/Rice |  |  |  |  |  |  |
| 13v.Pasteries/Cookies/Chips |  |  |  |  |  |  |
| 13w. Chocolate bars |  |  |  |  |  |  |
| 13x. Nuts or seeds |  |  |  |  |  |  |
| 13y. Ice cream |  |  |  |  |  |  |
| 13z. Table salt |  |  |  |  |  |  |

S1 Table Food frequency questionnaire: How often do you eat these foods?
